# *Cissus quadrangularis* L. extract attenuates chronic ulcer by possible involvement of polyamines and proliferating cell nuclear antigen

**DOI:** 10.4103/0973-1296.66941

**Published:** 2010

**Authors:** Mallika Jainu, K. Vijaimohan, K. Kannan

**Affiliations:** *Department of Biomedical Engineering, Sri Siva Subramaniya Nadar College of Engineering, SSN Nagar, Chennai - 603 110, India*; 1 *Department of Biochemistry, University of Madras, Guindy, Chennai - 600 025, India*

**Keywords:** *Cissus quadrangularis* extract, cytoprotection, polyamines, proliferating cell nuclear antigen

## Abstract

The present study was designed to investigate whether *Cissus quandrangularis* extract (CQE) had healing effects on gastric ulcer, through modulation of polyamines and proliferating cell nuclear antigen (PCNA) in rats. Administration of acetic acid (AA) was accompanied by reduced PCNA which was determined by immunohistochemical staining, ^3^H-thymidine incorporation using liquid scintillation spectrometry, mitochondrial marker enzymes, polyamine contents and transforming growth factor-alpha (TGF-α) expression in gastric mucosa of rats. Administration of CQE after the application of AA to the stomach enhanced the reduction of ulcer area in a dose-dependent manner which was confirmed by histoarchitecture. Moreover, CQE significantly increased the ^3^H-thymidine incorporation and the levels of polyamines such as putrescine, spermine and spermidine in ulcerated rats. In addition, the extract offers gastroprotection in the ulcerated area by increased expression of TGF-α and also reversed the changes in the gastric mucosa of ulcerated rats with significant elevation in mitochondrial tricarboxylic acid (TCA) cycle enzymes and PCNA levels. Based on these results, the healing effect of CQE on AA induced gastric mucosal injury in rats may be attributed to its growth promoting and cytoprotective actions, possibly involving an increase in tissue polyamine contents and cell proliferation.

## INTRODUCTION

Gastric ulcer, a common clinical entity, is treated by a variety of antisecretory and ulcer healing drugs. Many experimental investigations have been undertaken to elucidate the ulcer healing mechanism of various therapeutic drugs. Ulcer healing involves resolution of inflammation and repair of gastric tissues through granulation tissue formation, re-epithelialization and extracellular matrix remodeling.[1] Mucosal restitution is achieved by rapid migration of viable cells to the injured site and is finally accomplished by cell proliferation to replace the number of dead cells during ulcer healing.[2] Angiogenesis is a pivotal process in all types of wound healing, including the healing of gastric ulcers which is regulated by prostaglandins, proangiogenic factors, including vascular endothelial growth factor (VEGF) and transforming growth factors. The proliferative response of gastric mucosal cells to growth factors might be of special importance in maintaining gastric mucosal integrity and accelerating ulcer healing.[3]

Any potent antiulcer drug should stimulate mucosal proliferation, regeneration and polyamine synthesis, afford protection by attenuating the mitochondrial dysfunction, increasing the production of growth factors, healing of acute lesions and inhibition of gastric acid secretion.[4] Therefore, the link between cell proliferation, polyamines, growth factors and drug treatment may offer a new insight in ulcer therapy. Recently, various strategies have been adopted on the healing of chronic gastric ulcers clinically, and many adverse effects of these drugs have also been reported.

Currently, herbal medicines and foodstuff are the demand of the day as alternate source of modern medicine to cure gastric ulcer. *Cissus quadrangularis* Linn. (Vitaceae), commonly known as “bone setter”, is referred to as “Asthisamdhani” in Sanskrit.[5] This plant is frequently used as a common food item in India. This plant contains high amount of dietary antioxidants that include vitamin C, carotenoids and polyphenols.[6] Previous phytochemical studies have yielded triterpene, i.e., α-amyrin and α-amyrone, phytosterol, i.e., β-sitosterol, ketosteroid, oxo-steroid, two asymmetrical tetracyclic triterpenoids, namely, onocer-7-ene-3α, 21β-diol and onocer-7-ene3β, 21α-diol, stilbene derivatives, quercitin and calcium.[7] *C. quadrangularis* possesses analgesic, antiosteoporotic, anti-inflammatory, antimicrobial, hypolipidemic and antioxidant properties.[8–10] Due to its widespread health use and pharmacologic actions, this study will cherish the health promoting and therapeutic effect of *C. quadrangularis*.

We reported previously that *C. quadrangularis* extract (CQE) attenuated the deleterious effects of aspirin induced gastric mucosal damage through its antioxidant mechanism.[11] CQE is also known to stimulate cell proliferation, gastric mucus synthesis and secretion in indomethacin induced gastric ulcer model.[12]

Acetic acid (AA) induced gastric ulcer is pathologically similar to chronic gastric ulceration in humans.[13] Following our ethnopharmacologic studies on *C. quadrangularis*, further research is required to elucidate the ulcer healing mechanism of CQE on chronic gastric ulcer. This study aimed to investigate the effect of CQE on the epithelial cell proliferation, tricarboxylic acid (TCA) cycle enzymes, growth factors and polyamine levels in AA induced gastric ulcer in rats.

## MATERIALS AND METHODS

### Drugs

Trinitrobenezene sulfonic acid, AA, sucralfate (SFT), ethyldiamine tetraacetic acid (EDTA) and diaminobenzidine hydrochlorides were purchased from Sisco Research Lab (Mumbai, India). Antirabbit IgG goat conjugated with horseradish peroxidase (HRP) and antigoat IgG rabbit conjugated with fluorescein-5-isothiocyanate FITC were obtained from National Institute of Immunology (New Delhi, India). The radioactive compound,^3^H thymidine was obtained from Santa Cruz Biotechnology, Inc. (Santa Cruz, CA, USA).

### Plant material and extraction

The stem parts of *C. quadrangularis* used in this study were purchased from Native Care and Cure Center (Chennai, India) and identified with the standard sample in the Pharmacognosy Department, Captain Srinivasa Murthy Drug Research Institute for Ayurveda, Chennai, India. Dried stem parts of *C. quadrangularis* were coarsely powdered and 1 kg of this powdered plant material was soaked in 2 l of methanol for 48 h and the extract was filtered. This process of extraction was repeated and the filtrates were pooled and then distilled on a water bath. The last traces of the solvent were removed under vacuum drier and the solid brown mass obtained was stored at –4° C until further use. The yield of the extract for 1 kg of the plant material was 5.2%. For administration, 1 g of the extract was dissolved in 20 ml of distilled water and used for this study. All the test samples were administered by oral gavage in a volume of 1 ml/100 g body weight once daily to each rat.[12]

### Animals

Male albino Wistar rats weighing about 175-200 g were purchased from Tamil Nadu University of Veterinary and Animal Sciences (Chennai). The animals were housed at 27 ± 2°C temperature, 55% humidity, and a 12 h light/dark cycle. They were fed with standard laboratory chow (Hindustan Lever Foods, Bangalore, India) and provided with water *ad libitum*. Experimental protocols were approved by our institutional ethical committee, which follows the guidelines of Committee for the purpose of control and supervision of experimental on animals (CPSCEA) (IAEC no.: 01/027/04).

### Toxicity studies

For acute oral toxicity studies, rats were divided into four groups of six animals each. Group 1 served as control and received only distilled water, while groups 2, 3 and 4 were orally administered with CQE at dose levels of 1.5, 3.0 and 5.0 g/kg body weight, respectively, for 14 days. Rats were observed for signs of toxicity during the treatment period and on the 14th day, all the animals were sacrificed. Blood was collected by sinus puncture and analyzed for red blood cell count (RBC), white blood cell count (WBC), hemoglobin (Hb), hematocrit (HCT), mean corpuscular volume (MCV) and blood sugar using autoanalyzer, and blood urea was assessed by diacetyl monooxime (DAM) method.[14]

### Gastric ulcer induction in rats

Gastric kissing ulcers were induced by luminal application of AA as previously described.[13] Briefly, rats were starved for 24 h and their stomachs were exposed under ether anesthetization. The anterior and posterior walls of the stomach were clamped with a pair of metal rings of 11 mm internal diameter. Then, 0.12 ml of AA solution (60% v/v in distilled water) was injected into the clamped portion and withdrawn into the syringe after 45 s. The abdomens were then closed and the rats were allowed to recover with free access to food and water. One day after ulcer induction, rats were orally administered with one of two different doses of CQE (250 and 500 mg/kg body weight) in 1 ml of distilled water, and to another group of rats, SFT, a cytoprotective agent (500 mg/kg body weight) in 1 ml of distilled water was administered for 3 or 7 days, and then sacrificed by cervical dislocation on day 4 or day 8, respectively. Rats in the ulcer group were similarly treated with distilled water equivalent to the volume of extract solution. The stomach was removed and cut open along the greater curvature and size of lesions was measured as the sum of length of all lesions.[15]

### Experimental groups

Animals were divided into four groups of six animals in each group. Group 1 animals served as control that received only distilled water equivalent to the volume of plant extract. Group 2 represented the ulcerated group. Ulceration was produced by the administration of AA (60% v/v in distilled water) of 0.12 ml was injected (by the procedure mentioned above) and then similarly treated with distilled water equivalent to the volume of plant extract. Group 3 animals received AA (the same dose mentioned in group 2) and then the CQE (500 mg/kg body weight) was prepared with distilled water in a volume of 1ml and administered by oral gavage, once daily for 7 days. Group 4 animals received CQE (500 mg/kg body weight/ml) alone for 7 days orally and all the groups of rats were sacrificed the day after the last dose of drug treatment. Gastric mucosal tissues over the ulcer margin were removed by scrapping with a glass slide and immediately frozen in liquid nitrogen and stored at –70°C until determination for different parameters.

### Estimation of polyamines

The levels of polyamines in the gastric mucosal tissue were determined based on the method described by Endo.[16] The scrapped mucosal tissues were homogenized in ice-cold 0.4 M perchloric acid containing 2 mM EDTA and centrifuged at 3000 rpm for 5 min. Each sample solution (0.5–3.0 ml) was applied to CM-cellulose column (0.6 × 10 cm) equilibrated with phosphate buffer 0.01 M, pH 6.2. The putrescine, spermidine and spermine fractions were collected at a flow rate of about 3.0 ml/min and 1 ml of trinitrobenezene sulfonic acid TNBS was added to the eluate. Then, the absorbance at 420 nm was measured within 20 min. The levels of polyamines were expressed as nanomoles per gram tissue.

### ^3^H thymidine incorporation assay

Cell proliferation was evaluated by measuring the incorporation of ^3^H thymidine into DNA of gastric tissue. This experiment was done by the method of Tones *et al*.,[4] with modifications. To evaluate DNA synthesis in cells, 1 µCi/g body weight of ^3^H thymidine diluted in 0.9% saline was injected intraperitoneally (i.p) to the rats. The gastric mucosal tissue was homogenized with 2% citric acid and DNA was extracted following the method of Schneider.[17] The content was transferred into a scintillation vial containing 3.0 ml of liquid scintillation fluid and it was then vortexed. The amount of ^3^H thymidine incorporated was measured using liquid scintillation spectrometry on a beta counter (LS-6500; Beckman Instruments, Inc., USA) and expressed as counts per minute per milligram DNA.

### Determination of epithelial cell proliferation

Cell proliferation in gastric mucosa was determined by immunohistochemical staining of proliferating cell nuclear antigen (PCNA) as previously described.[18] Gastric mucosal tissues were immediately placed separately in freshly prepared 4% paraformaldehyde in phosphate buffered saline (PBS), 127 mM NaCl, 2.7 mM KCl, 8.1 mM Na_2_HPO_4_, 1.5 mM KH_2_PO_4_ (pH 7.4) containing 50 mM EDTA, at 4°C overnight, followed by standard paraffin embedding for 4 h and a tissue section of 5 µm thick was made with the help of automated microtome. Paraffin was removed in xylene and rehydrated by successive passing through decreasing concentrations of ethanol and then incubated with 0.3% H_2_O_2_ in methanol solution for 15 min to block the endogenous peroxidase activity. Then, the sections were soaked in citrate buffer (pH 6.0) and microwaved (940 W) for 1 min for antigen exposure. They were allowed to incubate with anti-PCNA monoclonal antibody (PC 10) (Santa Cruz Biotechnology, Inc) (1:100) overnight at 4°C and then washed with 0.01 M PBS (pH 7.4). Then the sections were incubated with biotinylated secondary antibody (National Institute of Immunology, New Delhi, India) for 30 min and subsequently with streptavidin-biotinylated HRP complex for another 30 min. The sections were stained with diaminbenzidine-H_2_O_2_ solution and counterstained with Mayer′s hematoxylin. They were then dehydrated in ethanol of ascending concentrations, followed by cleaning in xylene. The number of PCNA labeled cells was counted under a microscope (400×). An averaged number of PCNA labeled cells per gland was obtained from 10 axial gastric glands at the ulcer margins.

### Estimation of mitochondrial enzymes

The scrapped gastric mucosa was homogenized (5%) in 1.5 ml of 10 mM phosphate buffer (pH 7.8) containing 30 mM KCl in Teflon Potter-Elvehjem glass homogenizer. The homogenate was then centrifuged at 800 *g* for 10 min and used for the mitochondrial isolation. The mitochondria were isolated from gastric mucosal tissues by the method of Johnson and Lardy.[19] A 10% (w/v) homogenate was prepared in 0.05 M Tris-HCl buffer (pH 7.4) containing 0.25 M sucrose and centrifuged at 15,000 *g* for 5 min. The resultant mitochondrial pellet was then washed and resuspended in buffer for the following biochemical investigations. The activities of isocitrate dehydrogenase (ICDH),[20] α-ketoglutarate dehydrogenase (α-KGDH),[21] succinate dehydrogenase (SDH)[22] and malate dehydrogenase (MDH)[23] were assayed in mitochondria. The mitochondrial protein content was estimated by the method of Lowry *et al*.[24] Oxygen consumption was measured in a Clarke-type oxygen electrode as described by Katyare *et al*.[25] ADP/O ratio and respiratory control ratio (RCR) were calculated as described by Estrabrook.[26] The ATP content was estimated by the method of Williams and Corkey.[27]

### Histologic studies

The scrapped gastric mucosal tissues from each group were fixed in 10% formalin for 24 h. The fixative was removed by washing through running tap water overnight. After dehydration through graded series of alcohols, the tissues were cleaned in methyl benzoate and embedded in paraffin wax. Sections were cut into 3–5 µm thickness and stained with hematoxylin and eosin. After dehydration and cleaning, the sections were mounted and observed under light microscope for details.

### Immunohistochemical expression of transforming growth factor-alpha

Immunolocalization of transforming growth factor-alpha (TGF-α) in rat gastric mucosal tissue was carried out according to the “indirect peroxidase” method. The following procedure was done according to the method of Hsu and Rain.[28] Gastric mucosal tissues were fixed in 10% buffered formalin for 24 h. Paraffin blocks were made by embedding tissue for 4 h in an automatic block machine. Sections were cut into 5 µm and adhered to silanized slides (Sigma) and were dehydrated through xylene and alcohol series. After two rinses in PBS for 5 min each, the endogenous peroxidase activity was removed by incubation in 3% hydrogen peroxide for 30 min at room temperature. The non-specific binding sites were blocked by incubation with normal goat serum [three drops in 3% bovine serum albumin (BSA) in PBS] for 30 min. The sections were then incubated with primary antibody (anti Ib 1:250, anti II 1:100 and anti IIIa 1:100) for 60 min at room temperature. After rinsing with PBS, sections were incubated with biotinylated antiserum (goat antiserum to rabbit IgG 1:50 dilution) for 60 min at room temperature. Then, the sections were incubated in the working streptavidin HRP solution for 60 min at room temperature and washed thrice with PBS. Finally, the sections were incubated with diamine benzidine (DAB)-hydrogen peroxide for 30 min and washed in water, counterstained with Mayer′s hematoxylin and viewed under light microscope. Analysis of TGF-α product was performed by laser densitometry.

### Statistical analyses

The results were presented as mean ± SD. The data were also analyzed by one-way analysis of variance (ANOVA) using SPSS package. The statistical analysis was performed using Dunnett’s T3 multiple comparison test for all parameters. The values were considered significant at the levels of *P* < 0.05, *P* < 0.01 and *P* < 0.001.

## RESULTS

### Toxicity studies

The results of the oral administration of CQE in various doses of 1.5, 3.0 and 5.0 g/kg body weight for 14 days on different hematological and biochemical parameters in rats are presented in Table 1. Rats did not show any signs of toxicity or gross behavioral changes. The CQE treated rats even at the high dose of 5.0 g/kg body weight showed nonsignificant effect on RBC, WBC, Hb, HCT, MCV, blood sugar and urea as compared to the control group. The LD_50_ of *C. quadrangularis* was thus found to be more than 5 g/kg body weight.

**Table 1:** Toxicological evaluation of CQE in rats

Parameters	Control	CQE (1.5 g/kg body weight)	CQE (3.0 g/kg body weight)	CQE (5.0 g/kg body weight)
WBC (×103/µl)	7.42 ± 0.65	7.39 ± 0.35	7.32 ± 0.41	7.25 ± 0.39
RBC (×106/µl)	8.39 ± 0.32	8.54 ± 0.41	8.79 ± 0.49	8.91 ± 0.61
Hb % (g/dl)	12.38 ± 0.08	12.46 ± 0.09	12.53 ± 0.11	12.81 ± 0.11
MCV (fl)	43.51 ± 1.8	43.75 ± 2.3	43.98 ± 2.7	44.06 ± 3.2
HCT (%)	36.22 ± 1.9	36.39 ± 2.1	36.51 ± 2.6	36.84 ± 2.9
Sugar (mg/dl)	117.15 ± 3.7	117.40 ± 5.1	117.7 ± 5.9	118.61 ± 6.0
Urea (mg/dl)	16.73 ± 1.9	16.71 ± 1.4	16.23 ± 1.2	16.02 ± 1.5

The values are the means ± SD, *n* = 6; CQE treated groups showed nonsignificant changes when compared with control rats

### Effect of *C. quadrangularis* extract on gastric ulcer healing in rats

**Figure 1:**
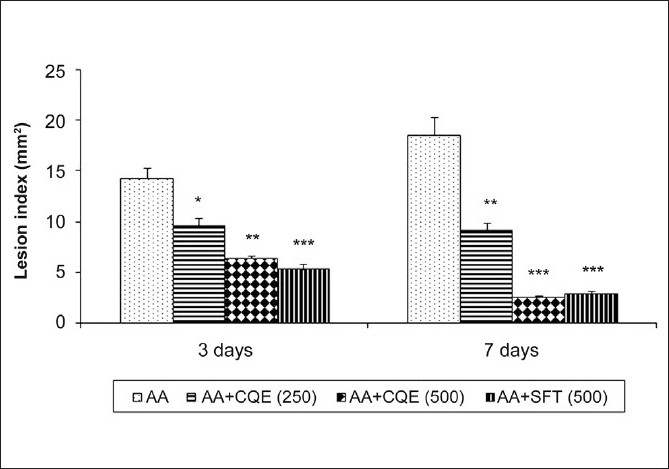
Effect of CQE on AA induced gastric ulcers in rats. Treatment with CQE decreased the number of ulcer lesions dose dependently and its healing effect is high at a dose of 500 mg/kg body weight for 7 days. The CQE protective ratio is nonsignificant when it is compared with that of standard ulcer healing drug, SFT. Values are expressed as mean ± SD for six animals in each group. *P < 0.05, ** *P* < 0.01,*** *P* < 0.001 statistically significant from ulcerated group (AA)

Figure 1 shows the healing effect of CQE on the area of gastric ulcers. AA induction to the stomach caused increased number of ulcer lesions in the mucosal area. Administration of CQE (250 and 500 mg/kg body weight) for 3 or 7 days was associated with a dose-dependent acceleration of ulcer healing at the ulcer margin. The protection index was high at a dose of CQE (500 mg/kg body weight) for 7 days and its ulcer healing effect was statistically insignificant in comparison to that of the reference drug, SFT. The dosage and duration of CQE treatment have been fixed for further studies based on the results of ulcer lesions scores.

### Effect of *C. quadrangularis* extract on gastric mucosal polyamine levels

**Figure 2:**
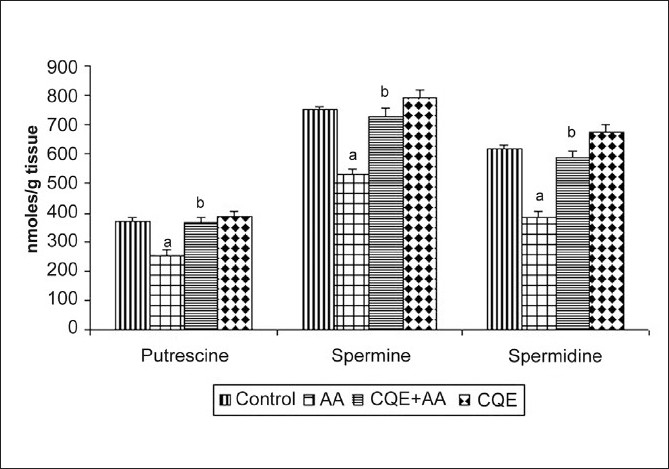
Effect of CQE on polyamine levels in the gastric mucosa of AA induced rats. Polyamines such as putrescine, spermidine and spermine were isolated from gastric mucosa by column chromatography and the levels were measured using spectrophotometer. Treatment with CQE increased the levels significantly as compared with AA induced rats. Results are expressed as mean ± SD for six animals in each group. ^a^*P* < 0.001 statistically significant as compared with control rats and ^b^*P* < 0.001 statistically significant as compared with AA induced rats

AA treatment caused a significant decrease in gastric mucosal putrescine, spermine and spermidine levels compared to control rats [Figure 2]. Treatment with CQE ameliorated the changes induced by AA administration and attenuated the decline in polyamine contents as compared to ulcerated rats. CQE administration alone caused a slight rise in polyamine contents but the changes were nonsignificant as compared with control.

### Effect of *C. quadrangularis* extract on ^3^H-thymidine uptake in the gastric mucosa

**Figure 3:**
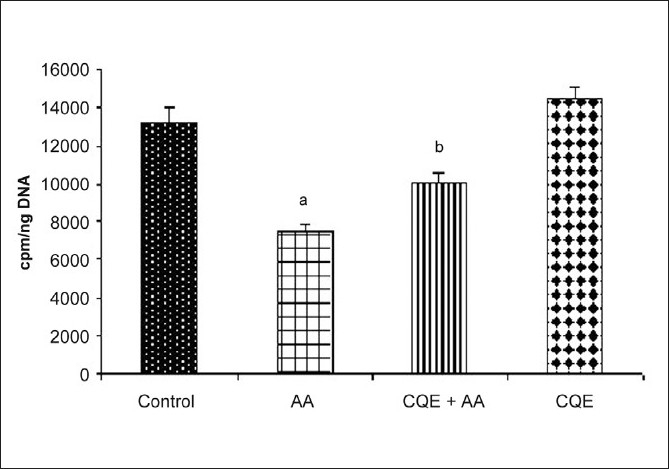
Effect of CQE on 3H-thymidine incorporation in the gastric mucosa of AA induced rats. Treatment with CQE (500 mg/kg body weight) increased the 3H-thymidine incorporation in gastric mucosa of AA induced rats, which was quantified by liquid scintillation counting. Results are expressed as mean ± SD for six animals in each group. ^a^*P* < 0.001 statistically significant as compared with control rats and ^b^*P* < 0.001 statistically significant as compared with AA induced rats

AA administration caused a sustained decrease in gastric mucosal ^3^H-thymidine incorporation compared to control rats [Figure 3]. On the contrary, a strong increase in ^3^H-thymidine uptake was observed in the gastric mucosa of CQE treated rats even after the exposure of AA. On the other hand CQE alone treated rats showed a slight rise in the radiolabeled incorporation of gastric mucosal portion as compared to control group.

### Effect of *C. quadrangularis* extract on epithelial cell proliferation

**Figure 4:**
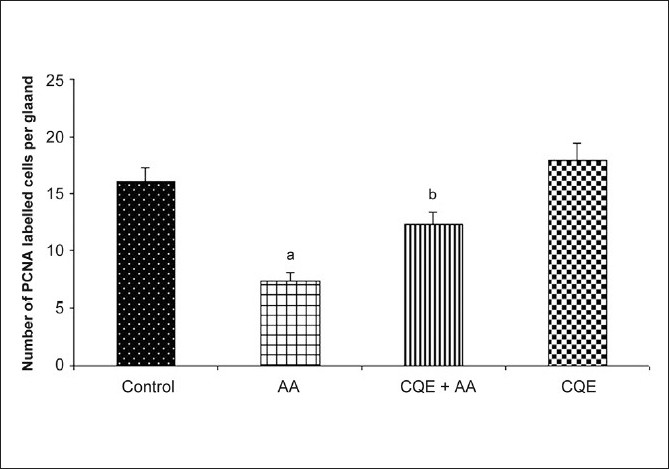
Effect of CQE on PCNA in AA induced rats. AA induced rats were treated with CQE (500 mg/kg body weight) for 7 days, the epithelial PCNAs in gastric mucosa was determined using immunohistochemical staining. Results are expressed as mean ± SD of six rats in each group. ^a^*P* < 0.001 statistically significant when compared with control rats and ^b^*P* < 0.001 statistically significant when compared with AA induced rats

We assessed the number of proliferating cells in gastric mucosal portion of CQE treated and untreated rats [Figure 4]. The results of the present study show that number of proliferating cells was significantly reduced in the ulcerated rats (group 2) than that of control (group 1). CQE increased the number of PCNA labeled cells at the ulcer margin on day 7 after ulcer induction when compared with the respective ulcerated group. CQE *per se* administered rats registered no significant change in the proliferative score in comparison with control.

### Effect of *C. quadrangularis* extract on mitochondrial enzymes and oxidative phosphorylation

Table 2 shows the activities of tricarboxylic acid (TCA) cycle enzymes such as ICDH, a-KGDH, SDH, MDH and changes in state 4, RCR, ADP/O and ATP levels in control and experimental groups of rats. There was a significant increase in the level of state 4 and a concomitant decrease in the activities of TCA cycle enzymes and in the level of RCR, ADP/O and ATP in ulcerated rats. CQE at a dose of 500 mg/kg body weight significantly showed minimal protection against mitochondrial dysfunction when compared with ulcerated rats, whereas no changes were seen in CQE treated and control group.

**Table 2:** Effect of CQE on the activities of TCA cycle enzymes and the levels of oxidation of succinate – state 4, RCR, ADP/O ratio and ATP content in the gastric mucosal mitochondria of AA induced rats

Groups	ICDH	α-KGDH	SDH	MDH	State 4	RCR	ADP/O	ATP
Control	317.6 ± 14.2	45.9 ± 3.6	266.8 ± 13.5	215.9 ± 14.5	18.7 ± 1.0	6.75 ± 0.35	2.38 ± 0.18	1.54 ± 0.12
AA	283.9 ± 10.5a′	41.1 ± 3.2a′	230.9 ± 11.6a′	184.8 ± 12.3a′	30.2 ± 2.4^a^	1.65 ± 0.10^a^	0.79 ± 0.04^a^	1.06 ± 0.09a′
AA + CQE (500 mg/kg body weight)	298.1 ± 12.3b′	43.9 ± 3.3	242.4 ± 14.8b′	202.1 ± 14.5b′	22.5 ± 1.5^b^	4.28 ± 0.33^b^	1.46 ± 0.11^b^	1.38 ± 0.10b′
CQE	311.6 ± 15.6^b^	45.1 ± 4.6b′	259.9 ± 15.6b′	213.6 ± 13.8^b^	18.3 ± 2.9^b^	6.23 ± 0.41^b^	1.99 ± 0.16^b^	1.43 ± 0.13^b^

The values are the means ± SD, *n* = 6, Enzyme activities are expressed as ICDH – nmoles of α-ketoglutarate formed/min/mg protein; α-KGDH – nmoles of potassium ferricyanide formed/min/mg protein; SDH – µmoles of succinate oxidized/min/mg protein; MDH – µmoles of NADH oxidized/min/mg protein; state 4 – nmoles of oxygen/min/mg protein; ATP – µg/mg protein,

a, a′ Statistically significant from control at *P* < 0.001 and *P* < 0.01

b, b′ statistically significant from AA administered group at *P* < 0.001 and *P* < 0.05

### Histologic studies of acetic acid induced ulcers

**Figure 5:**
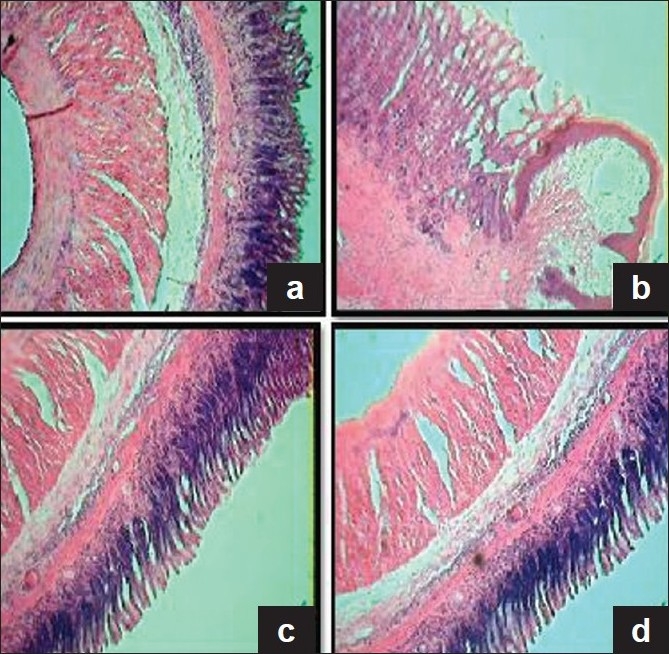
Histologic examination of gastric mucosal section in the control and experimental rats (H&E, 4×). (a) Section of gastric mucosa obtained from control rat, showing intact cellular architecture. (b) Section of gastric mucosa obtained from AA induced rat, showing marked ulcer crater and damage of submucosal layer, and muscularis region has been replaced by necrotic tissue. (c) Section of gastric mucosa obtained from CQE and AA administered rat, showing absence of ulcer crater, restitution of submucosal layers along with normal glands, complete clearing of inflammatory exudates and reepithelization. (d) CQE alone treated rat depicting normal appearance in the arrangement of mucosal layers almost similar to that of control rat

Histologic studies also provided supportive evidence for the biochemical analysis. Gastric mucosal surface of normal rats and CQE alone treated rats were intact with normal cellular architecture. Ulcerated rats showed sharply defined ulcer crater at the site of exposure to AA. Damaged mucosal epithelium, glands, leukocyte infiltration and cellular debris, ulcerated wall of the material covered with necrotic material in group 2 rats. Treatment with CQE showed absence of ulcer crater, clear differentiation of glandular structure, absence of necrosis, maintenance of polarity of epithelial cells in AA induced rats [Figure 5].

### Effect of *C. quadrangularis* extract on transforming growth factor-alpha expression in the gastric mucosa

**Figure 6:**
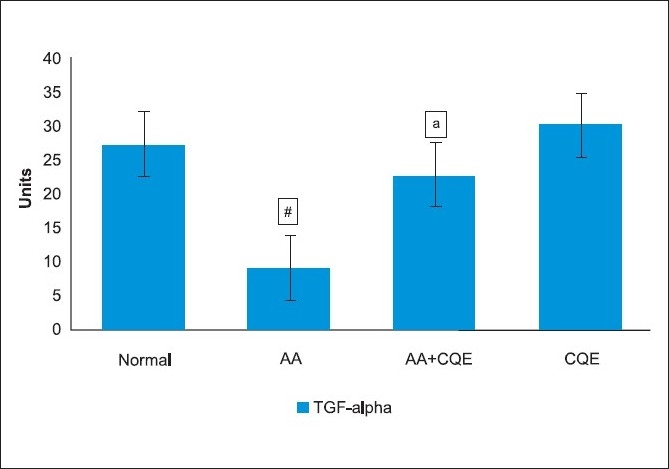
Results of laser densitometry for immunohistochemical expressions of TGF-α are shown in a bar graph. AA induced rat shows less expression of TGF-α than control rats. CQE TGF-α AA administered rat shows protection against gastric ulcers due to marked regeneration and increased expression of TGF-α. The results are presented as mean ± SD. ^a^Statistically significant from control at *P* < 0.001;^b^ statistically significant from AA group at *P* < 0.001

The expression of TGF-α was strong in the intact gastric mucosa of CQE alone treated rats (group 4) as in the control group (group 1). The expression of this factor was dramatically reduced in the ulcerated area of AA administered rats (group 2), while in CQE treated rats (group 3), a significant increase in mucosal expression of TGF-α was observed than that of ulcerated rats (group 2) [Figure 6].

## DISCUSSION

Ulcer healing consists of reconstruction of mucosal architecture and is a dynamic, active process of filling the mucosal defects with epithelial and connective tissue cells.[29] In order to promote tissue repair, induction of angiogenesis leading to microvascular reconstruction within the granulation tissue is also an important component in the healing mechanism. This study showed that AA induction significantly decreased the cell proliferation and polyamine levels by inhibiting angiogenic factors at the ulcer margin and base. The modern approach to control gastric ulceration is to promote gastroprotection, to block apoptosis and to stimulate epithelial cell proliferation for effective healing.

Toxicity studies carried out in rats indicate no lethal effect at least up to an oral dose of 5 g/kg body weight for 14 days, indicating that LD_50_, if any, should be higher than this dose. As reported recently, CQE administration at higher dose did not show any toxic effect on the vital organs as indicated by no significant difference in serum biochemical parameters.[12] Furthermore, in this study, hematological parameters also provided supportive evidence for the absence of adverse effects of CQE. In addition to other therapeutic properties of *C. quadrangularis*, it is also reported to possess antiulcer property on aspirin induced ulcer model by suppressing proinflammatory cytokines and free radical productions.[30] This study demonstrated that efficacy of the extract at the dose used was thus comparable to the ulcer healing effect of SFT (a nonsystematic ulcer healing drug), which caused about 90% of inhibition of kissing gastric ulcers,[31] since SFT prevents the delay in ulcer healing via the promotion of the granulation tissues and stimulates gastric epithelial proliferation in rats.

Polyamines are indispensable factors for normal repair of gastric and duodenal erosions, and they are known to participate in the progression of cell cycle followed by cell proliferation during ulcer healing.[32] Depletion of polyamine synthesis results in inhibition and/or apoptosis of mucosal cell proliferation, which may delay ulcer healing. Animal studies demonstrated that AA delayed ulcer healing as a result of reduction of cell proliferation by reducing growth promoting polyamines.[33] The excessive polyamine release appears to act as a mediator of CQE induced acceleration of healing of ulcer lesions due to its growth promoting actions.

AA is known to reduce DNA synthesis in the gastric mucosal epithelium, which served as the principle cause of the decreased proliferation, indicated by the reduced uptake of ^3^H thymidine by the cells.[4] Our findings suggest that CQE plays an important role in polyamine formation which triggers DNA synthesis as indicated by ^3^H thymidine incorporation in repair after gastric mucosal damage. We have recently observed that CQE could also prevent DNA fragmentation indicative of apoptotic cell death during gastric mucosal damage in rats. Ulcer healing is a complicated process of filling the mucosal defect with proliferation and migration of epithelial cells and stromal cells of connective tissues. This study indicates that CQE accelerates ulcer healing by enhancing mucosal repair and stimulation of cell proliferation in gastric epithelial cells. CQE may also stimulate the production of antioxidants at the wound site and provides a favorable environment for tissue healing, which might be due to its wound healing property.[12]

Mitochondrial dysfunction in ulcer induced group was evidenced by decreased activities of TCA cycle enzymes and energy production in this study, which corroborates with the work of Baracca *et al*.[34] Pretreatment with CQE ameliorated the changes in mitochondria in ulcerated rats by high availability of the substrate, which leads to the increase in these enzyme activities. Mitochondrial uncoupling is characterized by increased O_2_ consumption in state 4 and reduced RCR. The increase in oxygen consumption with a significant increase in state 4 and significant reduction of RCR, ADP/O ratio and ATP content upon AA administration would result in inhibition of oxidative phosphorylation in mitochondria,[35] which corroborates with the present study. CQE displayed gastroprotection at the mitochondrial level by reducing O_2_ consumption in state 4 thereby leading to increase in RCR and ADP/O ratio this could be due to increased mitochondrial enzymes activities in the gastric mucosa of ulcerated rats.

Histologic studies showed that AA inhibited tissue contraction and regeneration of the ulcerated mucosa. Complete regeneration of mucosal glandular structures of CQE was well evidenced through histologic studies. Cell migration and cell proliferation are the two important processes responsible for the early phase of gastric ulcer healing, which are markedly enhanced by CQE.

Mucosal growth is under the influence of various growth factors, among which TGF-α seems to play a crucial role. TGF-α that is produced in the gastric mucosa contributes to the healing of AA induced ulcers in rats by the enhancement in cell proliferation.[36] The increased production of TGF-α stimulates mucosal regeneration, polyamine synthesis, protein synthesis, heals acute lesions and inhibits gastric acid secretion. The constituents, such as quercitin and triterpenoids, present in CQE are also possible TGF-α stimulators, which may be responsible for the cytoprotective action.[3,37]

However, the main components of *C. quadrangularis*, such as triterpenoids, vitamin C,[38] β-sitosterol, glycosides and polyphenols, offer antiulcer and ulcer healing properties.[39] Vitamin C was also found to increase collagen synthesis in wound tissues and stimulate cell growth.[3] The combined effect of these constituents in CQE could have been more responsible for the tissue repair in AA administered rats. This result implicates that the tissue repair stimulatory action of CQE at the ulcer site may be mediated through excessive release of polyamines and promotes angiogenesis via TGF-α.

In conclusion, the mechanism of ulcer healing effects of CQE involves the synthesis of polyamines, which play a crucial role in the growth promoting action of TGF-α, cell proliferation and it also prevents uncoupling of oxidative phosphorylation. Together, these findings merit detailed analysis relating to active principles of CQE and the mechanism of action, which might provide new alternatives for the clinical management of gastric ulcer diseases. Furthermore, it may be suggested that since *C. quadrangularis* is a commonly consumed diet in India, the results may help to provide scientific basis for home remedy for the treatment of ulcers.
